# Simplified data access on human skeletal muscle transcriptome responses to differentiated exercise

**DOI:** 10.1038/sdata.2014.41

**Published:** 2014-11-25

**Authors:** Kristian Vissing, Peter Schjerling

**Affiliations:** 1 Section of Sport Science, Department of Public Health, Aarhus University, Aarhus DK-8000, Denmark; 2 Institute of Sports Medicine, Department of Orthopaedic Surgery M, Bispebjerg Hospital and Center for Healthy Aging, Faculty of Health and Medical Sciences, University of Copenhagen, Copenhagen DK-2400, Denmark

**Keywords:** Biomarker research, Skeletal muscle, Transcriptomics

## Abstract

Few studies have investigated exercise-induced global gene expression responses in human skeletal muscle and these have typically focused at one specific mode of exercise and not implemented non-exercise control models. However, interpretation on effects of differentiated exercise necessitate direct comparison between essentially different modes of exercise and the ability to identify true exercise effect, necessitate implementation of independent non-exercise control subjects. Furthermore, muscle transcriptome data made available through previous exercise studies can be difficult to extract and interpret by individuals that are inexperienced with bioinformatics procedures. In a comparative study, we therefore; (1) investigated the human skeletal muscle transcriptome responses to differentiated exercise and non-exercise control intervention, and; (2) set out to develop a straightforward search tool to allow for easy access and interpretation of our data. We provide a simple-to-use spread sheet containing transcriptome data allowing other investigators to easily see how mRNA of their gene(s) of interest behave in skeletal muscle following exercise, both endurance, resistance and non-exercise, to better aid hypothesis-driven question in this field of research.

## Background & Summary

Metabolic stress inherent of endurance exercise versus mechanical stress inherent of resistance exercise is considered to comprise essentially different stimuli that drive training-induced muscle adaptations towards opposite ends of an adaptation continuum. According to this traditional contention, increased capability for oxidative metabolism is induced by endurance training, whereas increased capability for contractile force development is induced by resistance training^1–3^.

The underlying myocellular mechanism involved in promoting exercise-specific adaptations is receiving substantial research attention, not least because exercise provide a highly relevant model to investigate mechanisms involved in clinical conditions affecting muscle metabolism or muscle contractile function, as well as counteracting sarcopenia in aging.

Studies on muscle phenotype regulation primarily consist of hypothesis-driven studies on pre-selected myocellular pathways, in attempts to tie specific regulatory pathways to specific types of skeletal muscle adaptations^4–12^. However, such studies are limited by not being able to comprise the potential effects of simultaneously cross-talking mechanisms, redundant mechanisms and/or oppositely acting mechanisms.

To provide a more complete overview on exercise-induced myocellular responses, in more recent years, omic´s-based techniques have been utilized and data have become available on exercise-induced transcriptome responses from studies on endurance exercise^13–16^, as well as from studies on resistance exercise^17–20^.

Valuable information can be retrieved from such studies, but certain aspects still render interpretation difficult. Firstly, all of the studies conducted on acute transcriptome responses to exercise have focused only on one specific type of exercise. However, oppositely to an traditional contention, exercise-induced muscle phenotype adaptations are likely dispersed along a continuum, as dictated by the physical condition of included subjects and by the choice of intensity, duration, volume, frequency and/or interspaced recovery of a given exercise protocol^1–3^. If exercise-induced adaptations disperse along a continuum, the responses of underlying myocellular regulatory mechanisms likely do the same, with some mechanism potentially responding essentially different to differentiated exercise, whereas others may exhibit overlap in responses. Such information is important, in order to manipulate specific mechanisms, while simultaneously avoiding unintended effects. Thus, investigation of this requires comparative investigation of endurance exercise versus resistance exercise, practised by commonly practised principles, but has not yet been conducted in humans. Furthermore, previously conducted studies include either untrained or well-trained individuals^13–20^. However, unfamiliar exercise may comprise an unrealistic type and/or magnitude of stress and well-trained athletes may exhibit very low sensitivity to familiar exercise. Investigation on moderately trained individuals can therefore be argued to mimic a more probable situation.

Another challenge in interpretation of exercise-induced myocellular responses relate to whether they can be even be regarded as genuine effects of exercise or if they are rather related to other confounding factors inherent of an exercise trial. In accordance, factors such as dietary condition, clinical procedures (i.e. tissue collection) and/or circadian rhythm, may all obscure interpretation^21^. Such knowledge requires non-exercise control experiments, yet nearly all studies on exercised-induced transcriptome responses have not.

Finally, although transcriptome data are now in principle available from exercise studies, the formats of data sets, makes them difficult to extract and therefore interpret by researchers inexperienced with the procedures and the flaws of bioinformatics. For instance, reported data most often rely on highest ranking of response based on an arbitrary cut-off level. While this may serve to highlight molecules that are highly responsive, it is not necessarily telling of biological importance. Oppositely, potentially important molecules responding below a pre-set cut-off level are easily ignored. Presentations based on degree of responsiveness are not optimal to illustrate effects on related molecular family members or molecules related to the same pathway. Pathway tools exist to obtain such information, but can still be difficult to interpret. Development of procedures to allow more simplified unravelling of omic´s data can therefore be of great value.

Based on these challenges, one major aim of the current study was to compare exercise-induced human skeletal muscle transcriptome responses to differentiated exercise in training-accustomed individuals as well as towards non-exercise control intervention. This approach involved 10 weeks of prior endurance or resistance training to familiarise subjects before conducting the actual single-bout exercise trial to examine exercise-induced responses. Another major aim of the study was to develop a simplified procedure to aid search and interpretation of our data.

## Methods

### Subjects

The subjects have previously been described in details^22–26^. In brief, fourteen young untrained healthy male subjects were randomly divided into two groups; an endurance training group and a resistance training group. A further six subjects composed the non-exercising control group. All subjects were informed about the purpose and the risks related to the study and gave written, informed consent to participate. The study was approved by the ethics committee of Region Midtjylland (j.no. M-20080177) and performed in accordance with the Declaration of Helsinki.

### Experimental design and training protocol

**Figure 1:**
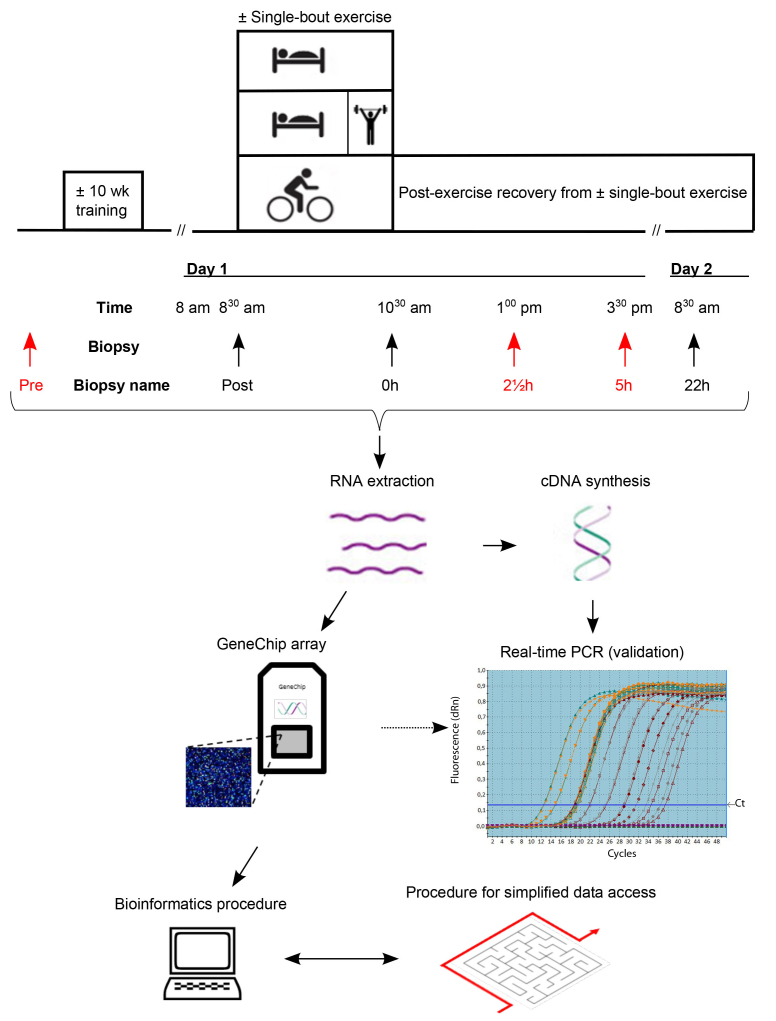
Experimental design. Three individual groups of human subjects had a biopsy taken prior to commencing 10 weeks of intervention (Pre). They then completed 10 weeks of intervention as either endurance, resistance or no training. After several days of recovery, subjects reported at the same time point of the day to the laboratory for±single-bout exercise, in which they conducted either endurance exercise (i.e., 2 h of cycling at 60% aerobic intensity), resistance exercise (i.e., rested for 1$\frac{1}{2}$ h followed by a 30 min session including 4 sets x 12 repetitions for three leg exercises) or no exercise (i.e., the subjects rested for 2 h). Biopsies were harvested at the same absolute time points prior to (i.e. similar to post training) and at 0, 2$\frac{1}{2}$, 5 and 22 h post±exercise. RNA was extracted and RNA from time points corresponding to Pre, 2$\frac{1}{2}$ and 5 h (time points for array analysis marked in red in the graph) were hybridized to GeneChip arrays followed by bioinformatics procedures and development of a search tool to allow simplified data access. In parallel, RNA from all time points were included in cDNA synthesis to be used for validation of array data by Real-time PCR.

The design and training protocols have previously been described in details^22–26^. In brief, prior to a single-bout exercise trial aiming to study acute gene expression responses, subjects were habituated through a prior 10 week training phase. At least three day before commencing this training phase, a biopsy was harvested (i.e. termed ‘Pre’, identical to pre training and used as pre value for investigation of acute transcriptome responses to single-bout exercise—see Figure 1 and further below in text). Exercise groups completed either 10 weeks of progressive endurance training (ET) on a cycle ergometers or 10 weeks of progressive conventional resistance training (ST) for lower extremity muscle groups. ET was performed on stationary bicycles (Kettler Ergoracer GT, Kettler, Ense-parsit, Germany). 3 weekly sessions consisted of one session each of continuous cycling of 30 to 45 min at 60–75% of Watt-max, a second session consisting of two intervals of 20 min at 70–80% of Watt-max, and a third session consisting of 8×4 min intervals at 80–90% of Watt-max. The RT group completed a conventional progressive overload training program, similar to that previously described^27^, consisting of three leg exercises each performed as 3–5 sets of 10→6 repetitions with repetitions corresponding to RM loading. All training sessions were supervised to ensure proper progression for both groups.

Following three days of recovery from the final exercise session of the training phase, subjects completed a single-bout trial including either the type of exercise they were habituated to through prior training or non-exercise for the control subjects. During this trial, muscle biopsies were harvested and used for transcriptome analysis.

### Single-bout exercise protocol

A schematic overview of the single-bout trial design is seen in Figure 1. Subjects arrived after an overnight fast at 8 am on day 1 for pre-exercise measurements. The subjects rested in the supine position for approximately 30 min. A pre-exercise muscle biopsy (i.e. termed ‘Post’, identical to post training) was then taken from the vastus lateralis muscle. At 8.30 am, subjects of the endurance group commenced 120 min of bicycle exercise at 60% post training Vo_2_ peak. Subjects in the resistance group remained resting until 10 am before commencing 4 x post training 12 RM of three thigh muscle exercises with 1$\frac{1}{2}$ minutes rest between sets, thereby allowing them to finish at 10.30 am (i.e. same absolute time point as for subjects of the endurance group). The three thigh muscle exercises were leg press, knee extension, and hamstring curl. Minor weight adjustments were allowed to ensure performance of 12 RM as precise as possible. Non-exercise control subjects rested from 8.30 am to 10.30 am. The non-exercise control group was included, to control for potential non-exercise related effects of fasting conditions and the invasive procedures inherent of the protocol. To control for potential effects of circadian rhythm, it was attempted to strictly equalise absolute daily time points as well as time resolution of the protocol, regardless of which group the subjects were assigned to. After±exercise, all subjects rested under fasting conditions (with water offered *ad libitum*) until 3.30 pm (corresponding to 5 h post exercise). They were allowed normal dietary intake for the remainder of day 1 and instructed to fast overnight before arriving in fasting condition to the laboratory on the morning of day 2 of the trial. During post-exercise recovery, muscle samples were harvested at 0, 2$\frac{1}{2}$, 5 and 22 h after exercise. All biopsies were harvested from separate incision holes and dispersed between legs.

### Preparation of muscle biopsies and RNA isolation

Biopsy samples harvested during the single-bout exercise trial were rinsed from visible fat and connective tissue and frozen in liquid nitrogen as quickly as possible. All samples were then stored at −80 °C until further investigation. Total RNA (from approximately 20 mg of muscle) was extracted using the guanidinium thiocyanate–phenol–chloroform extraction method as previously described^28^ and the concentration was determined spectrophotometrically using a Nanodrop 1000 (Thermo Fischer Scientific, Wilmington, DE, USA). RNA integrity was ensured by gel electrophoresis.

### Microarray

From six subjects in each of the three exercise groups, samples from the three time points, Pre (i.e. pre training), 2$\frac{1}{2}$ h and 5 h, were selected for microarray analysis (54 samples). 10 μl total RNA (2.5—8 μg) was purified further using the RNeasy MinElute Cleanup kit (Qiagen). The purified RNA was amplified and labeled using the Ambion WT Expression Kit (Applied Biosystems) according to manufactures instructions. 100 ng total RNA was used as input. The labeled samples were hybridized to the Human Gene 1.0 ST GeneChip array (Affymetrix, Santa Clara, CA, USA). The arrays were washed and stained with phycoerytrin conjugated streptavidin (SAPE) using the Affymetrix Fluidics Station® 450, and the arrays were scanned in the Affymetrix GeneArray® 3000 scanner to generate fluorescent images, as described in the Affymetrix GeneChip® protocol. Cell intensity files (CEL files) were generated in the GeneChip® Command Console® Software (AGCC) (Affymetrix, Santa Clara, CA, USA).The CEL files were analyzed using Partek Genomic Suite v6.6 (Partek), using the default Partek settings (GC content pre-adjustment, RMA backgrounds correction, quantile normalization, mean probe set summarization) to generate transcript expression levels (Data Citation 1). To test for unintended group differences, an ANOVA for Exercise was performed on the Pre samples only. No transcripts came out with a FDR<0.05. To facilitate comparison of changes over time all transcript expression values were normalized to their respective pre value for each subject. A repeated measures two-way ANOVA for Time, Exercise and Time x Exercise (REML for Variance Component Estimation) was performed to generate p-values for overall effects as well as between individual subgroups (Contrasts). Transcripts exclusively induced in a single group were identified as follows. The transcript level has to be significantly different from both Pre and the two other groups for at least one of the two time points. Furthermore, the transcript must not be significantly different from Pre at any time in the other groups. Time regulated transcripts were identified as follows: Time x Group (raw *P*>0.1) and Time (FDR<0.05) at 2.5 h or 5 h and all three groups should be significantly different from Pre (raw *P*<0.05) at the same time point. FDR (False discovery rate) of less than 0.05 is used as significance level unless stated otherwise.

### Real-time PCR

500 ng total RNA was converted into cDNA in 20 μl using the OmniScript reverse transcriptase (Qiagen, California, USA) and 1 μM poly-dT (Invitrogen, Naerum, Denmark) according to the manufacture's protocol (Qiagen). For each target mRNA, 0.25 μl cDNA was amplified in a 25 μl SYBR Green polymerase chain reaction (PCR) containing 1×Quantitect SYBR Green Master Mix (Qiagen) and 100 nM of each primer (Table 1). The amplification was monitored real time using the MX3005P Real-time PCR machine (Stratagene, California, USA) with the amplification protocol; 95°,15′->{95°,15′′->58°,30′′->63°,90′′(signal)}x50->melt curve analysis. The Ct values were related to a standard curve made with known concentrations of cloned PCR products or DNA oligonucleotides (UltramerTM oligos, Integrated DNA Technologies, Inc., Leuven, Belgium) with a DNA sequence corresponding to the sequence of the expected PCR product. The specificity of the PCR products was confirmed by melting curve analysis after amplification. The large ribosomal protein P0 (RPLP0) mRNA was chosen as internal control.

## Data Records

### Identification of regulated transcripts

To identify exercise induced mRNA, a microarray comparison was performed using the Pre samples and the two acute samples obtained during recovery from the single-bout trial, 2$\frac{1}{2}$ h and 5 h. Both acute changes within the first hours after exercise as well as long-term training changes would be caught by comparing the 2$\frac{1}{2}$ h and 5 h Post expression values with the Pre value. Six subjects from each exercise group were analysed using the Affymetrix Gene ST 1.0 arrays (28869 transcripts) (Data Citation 1). As the values of interest here are the changes from Pre, all expression values were normalized to the respective pre value before further analyses. Two-way repeated measures ANOVA shows that around a thousand mRNA were significantly changed in both the resistance and the endurance groups, whereas a much lower number were changed in the control group (Table 2). Comparisons between the groups within the two Post exercise time points also show massive differences between the groups, including endurance versus resistance, indicating large and different impact of endurance and resistance exercise on the transcriptome (Tables 3 and 4).

Several hundred transcripts can be identified as specifically responsive to a single exercise type, especially in the resistance training group (Table 5).

About one hundred transcripts can be identified as generally responding to exercise, independent of the exercise type, whereas, using very stringent criteria, 36 can be found to respond to both exercise type, but in different ways (Table 6).

The finding of some changes in the control group indicates a general time dependent effect as well, e.g. from circadian rhythm or dietary status. In principle such transcripts are those that display a significant time effect, but not an interaction, in the ANOVA. However, since 2/3 of the samples are exercise samples, a general exercise effect might erroneously also give such an effect, due to the relative low number of control samples compared to the exercise samples. To exclude those, a stringent criterion was added, such that furthermore, all three groups should have a raw *P*-value of less than 0.05 compared to Pre at the specific time point. By these criteria, 192 time regulated transcripts were identified, 131 at 2.5 h and 103 at 5 h (Table 7).

## Technical Validation

To validate the microarray data, we tested the expression of a few differentially expressed mRNA by another technique, real-time RT-PCR. In this case we included all time points to also test time points which were not included in the statistical analysis of the microarrays, i.e. outside potential selection bias.

**Figure 2:**
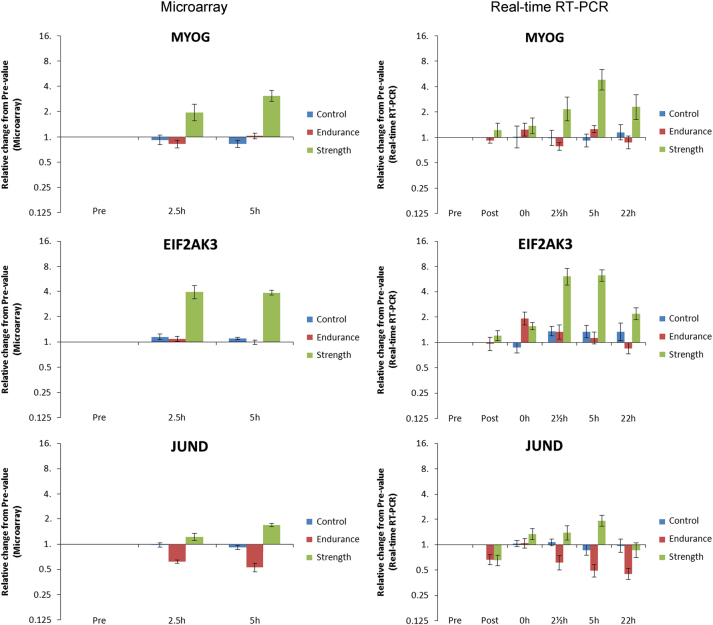
RT-PCR. Comparison of results obtained from the microarray data with results obtained using standard real-time RT–PCR. Only time-points Pre and 2$\frac{1}{2}$ and 5 h Post final exercise session are measured with microarrays (Left). All time-points were measured with real-time RT-PCR (Right). MYOG (Myogenin) and EIF2AK3 (eukaryotic translation initiation factor 2-alpha kinase 3) were identified as strength specific and JUND (Jun D) as exercise specific, but dependent on exercise type. Data are shown as geometric mean±back-transformed s.e.m. on a logarithmic axis (log2).

As can be seen in Figure 2 RT–PCR, there is a good correspondence between the results obtained with the microarray data and the results using real-time RT-PCR. Furthermore, the pattern is replicated in some of the time points not included in the microarray analysis, indicating that this is not a statistical artifact (type II error).

## Usage Notes

The data from this transcriptome analysis is summarized in an Excel file which can be downloaded from the GEO database (Data Citation 1).

The file contains five sheets containing instructions, statistical analysis and mean data, individual data, and two presentation sheets for mean and individual data, respectively.

The intention is that the reader can generate a list of all mRNA regulated by a specific condition or investigate the regulation of one or more specific mRNA by using the filtering function on the statistics or the names, respectively. Bar graphs or dot plots can then be easily generated by tapping in the unique ID’s in the two graph sheets.

**Figure 3:**
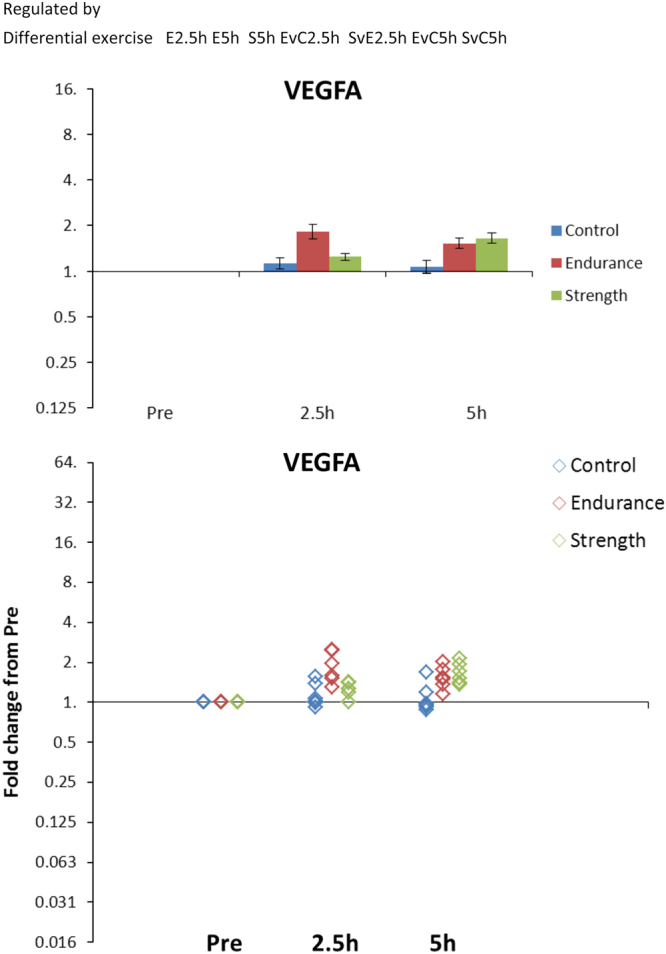
Example. Illustration of the graphs that can easily be generated for a specific mRNA using the Excel file. In this case the VEGFA mRNA. Besides getting the graphs, the statistical result is also provided, here showing that VEGFA mRNA is differentially regulated by exercise (Differential Exercise) and that within the Endurance group both 2.5 and 5 h are significantly different from Pre (E2.5h and E5h), whereas for the Strength group only 5 h is significant (S5h). Furthermore, within 2.5 h Endurance is different from both Control (EvC2.5h) and Strength (SvE2.5h), whereas at 5 h Endurance is only significantly different from Control (EvC5h) but not Strength (no SvE5h). On the other hand also Strength is different from Control at 5 h (SvC5h).

Figure 3 Example shows an example of the graphs generated for the VEGFA (Vascular Endothelial Growth Factor A) mRNA simply by writing ‘VEGFA’ in the two graph sheets.

## Additional information

**How to cite this article:** Vissing, K. & Schjerling, P. Simplified data access on human skeletal muscle transcriptome responses to differentiated exercise. *Sci. Data* 1:140041 doi: 10.1038/sdata.2014.41 (2014).

## Supplementary Material



## Figures and Tables

**Table 1:** Primers for real-time RT-PCR.

**mRNA**	**Genbank**	**Sequences (sense/antisense)**
RPLP0	NM_053275.3	GGAAACTCTGCATTCTCGCTTCCT/CCAGGACTCGTTTGTACCCGTTG
JUND	NM_005354.5	CGAGTCCACATTCCTGTTTGTAATCCT/GAAAACAGAAAACCGGGCGAAC
MYOG	NM_002479.5	CTGCAGTCCAGAGTGGGGCAGT/CTGTAGGGTCAGCCGTGAGCAG
EIF2AK3	NM_004836.5	TCAGCACTCAGATGGAGAGAGTCAGG/CTTGAACCATCACGTACTCACAAGGA

**Table 2:** Number of changed mRNA versus Pre.

**Group**	2$\frac{1}{2}$h	**5 h**
Control	44	43
Endurance	1,024	853
Resistance	1,324	1,840

**Table 3:** Number of changed mRNA between groups at 2½ h.

**Group**	**Control**	**Endurance**	**Resistance**
Control	—	1,132	971
Endurance	1,132	—	1,255
Resistance	971	1,255	—

**Table 4:** Number of changed mRNA between groups at 5 h.

**Group**	**Control**	**Endurance**	**Resistance**
Control	—	1,226	1,798
Endurance	1,226	—	1,643
Resistance	1,798	1,643	—

**Table 5:** Number of changed mRNA versus Pre specific to only one group (FDR <0.05).

	**Control**	**Endurance**	**Resistance**
Group specific	11	275	583

**Table 6:** Number of changed mRNA versus Pre in both exercise groups (FDR <0.05).

	**Independent of exercise type**	**Dependent of exercise type**
Group specific	107	36

**Table 7:** Number of changed mRNA versus Pre unrelated to exercise (FDR <0.05).

**Group**	2$\frac{1}{2}$ h	**5 h**
All	192	131
